# The Role of Frozen Section in Surgical Staging of Low Risk Endometrial Cancer

**DOI:** 10.1371/journal.pone.0021912

**Published:** 2011-09-01

**Authors:** Sanjeev Kumar, Sudeshna Bandyopadhyay, Assaad Semaan, Jay P. Shah, Haider Mahdi, Robert Morris, Adnan Munkarah, Rouba Ali-Fehmi

**Affiliations:** 1 Section of Gynecologic Surgery, Mayo Clinic, Rochester, Minnesota, United States of America; 2 Department of Pathology, Wayne State University, Detroit, Michigan, United States of America; 3 Division of Gynecologic Oncology, Department of Obstetrics and Gynecology, Wayne State University, Detroit, Michigan, United States of America; 4 Division of Gynecologic Oncology, Southern California Medical Group-Orange County, Orange County, California, United States of America; 5 Department of Obstetrics and Gynecology, University of Washington School of Medicine, Seattle, Washington, United States of America; 6 Division of Gynecologic Oncology at Henry Ford Health System, Department of Obstetrics and Gynecology, Detroit, Michigan, United States of America; Bauer Research Foundation, United States of America

## Abstract

**Background:**

The role of frozen section (FS) in intraoperative decision making for surgical staging of endometrial cancer is controversial. Objective of this study is to assess the agreement rate between the FS and paraffin section (PS); and the potential impact of the role of FS in the intra-operative decision making for the complete surgical staging in low risk endometrial cancer.

**Methods:**

This is a retrospective analysis of patients diagnosed with intra-operative FS stage I, grade I or II endometrial cancer from 1995–2004. FS results were compared with final pathology results with regard to tumor grade, depth of myometrial invasion, cervical involvement, lymphovascular invasion, and lymph node involvement. Agreement statistic with kappa was calculated using SPSS statistical software. Categorical variables were tested using chi-square test with p value of ≤0.05 being statistically significant.

**Results:**

Of the 457 patients with endometrial cancer, 146 were evaluated by intra-operative FS and met inclusion criteria. FS results were in disagreement with permanent section in 35% for the grade (kappa 0.58, p = 0.003), 28% for depth of myometrial invasion (kappa 0.61, p<0.0001), 13% for cervical involvement (kappa 0.78, p = 0.002), and 32% for lymphovascular invasion (kappa 0.6, p = 0.01). Permanent pathology upstaged 31.9% & 23.2% of FS stage IA, & IB specimen respectively. Lymph node dissection was done in 56.8%. Lymph node metastasis was identified in 8.4%. Use of intraoperative FS would have resulted in suboptimal surgical treatment in 13% stage IA and 6.6% of stage IB patients respectively by foregoing lymphadenectomy.

**Conclusion:**

A significant number of patients with low risk endometrial cancer by FS were upstaged and upgraded on final pathology. Before placing absolute reliance on intraoperative FS to undertake complete surgical staging, the inherent limitation of the same in predicting final stage and grade highlighted by our data need to be carefully considered.

## Introduction

Endometrial cancer is the most common gynecologic malignancy in the United States, with an estimated 43,400 new cases diagnosed and 8000 deaths annually [1]. Most patients (71%) have disease confined to the uterus (FIGO stage I) [1] [2]. In 1988 the International Federation of Gynecology and Obstetrics (FIGO) replaced clinical staging with surgical staging system for endometrial cancer [2]. It is supported by several studies which indicate that as many as 25% and 50% of patients with clinical stage I, or II disease respectively had disease outside the uterus at the time of comprehensive surgical staging [3].

Whereas there is general agreement about the necessity of complete surgical staging for high risk endometrial cancer as the risk of nodal metastasis is high [4]; the need for pelvic and paraaortic lymphnode dissection with complete surgical staging for the low risk endometrial cancer has been debated passionately. [5] [6] [7], [8]. The pendulum swings with some advocating only hysterectomy and bilateral salpingooophorectomy without node dissection for low risk endometrial cancer [9] while others advocating comprehensive surgical staging for all patients with low risk disease [10]. Still others take an intermediate path and believe that only a small fraction of patients with low risk endometrial cancer may benefit from routine and comprehensive surgical staging including a lymphadenectomy and the rest may be adequately managed by routine hysterectomy with bilateral salpingooophorectomy [11]. The key question however, is how best to identify these patients who have seemingly low risk endometrial cancer but may need complete surgical staging instead. One widely used approach to address this critical question is the use of intraoperative frozen section (FS) in the decision making process. Here, the surgeon completes a hysterectomy and if the FS shows high risk features, such as high grade, deep myometrial invasion, lymphovascular space invasion, adnexal or cervical involvement; then a comprehensive surgical staging is undertaken and vice versa. This approach is not without its pitfalls. The intra-operative assessment of grade and myometrial invasion is based on a limited sample and may not be in agreement with the final pathology. In addition, obscuring frozen artifact and interobserver variability of gross tumor evaluation would also confound the intraoperative microscopic assessment. It is therefore important to find the agreement rate of the FS with respect to its prediction of the final pathology in the paradigm of the complete surgical staging of the low risk endometrial cancer. The literature on this issue so far is controversial with some suggesting FS to be reliable [12] [13] whereas others refuting the same [14]. This controversy was highlighted by a recent study by Soliman et. al. where half of the physicians indicated that they do not use FS and the rest indicated that they use FS in their practice to decide when to perform lymphadenectomy in endometrial cancer [15]. Therefore further data is urgently needed to resolve the controversy in defining the role of FS in surgical staging of low risk endometrial cancer.

The primary aim of this study is to assess the agreement rate between FS and paraffin section (PS) in determining the grade, depth of myometrial invasion, cervical involvement and lymphovascular space involvement. The secondary aim is to assess the impact of disagreement between the FS and PS on the FIGO stage designation of patients with presumed stage I low-grade endometrial cancer by FS.

## Materials and Methods

### Ethics statement

This study was approved by the institutional review board (IRB) of the Wayne State University. No patient consent was required because the data were analyzed anonymously in a deidentified fashion and it was a retrospective study. The institutional review board (IRB) of the Wayne State University specifically waived the need for consent.

This is a retrospective review of endometrial cancer patients treated at Wayne State University from 1995 to 2004. All the patients had pre-operative diagnosis of low-grade endometrial cancer by endometrial biopsy or curettage. Here, low grade refers to FIGO grade I and II. FIGO staging used in the paper refers to the FIGO 1988 system of surgical staging.

Primary treatment was exploratory laparotomy with total abdominal hysterectomy and bilateral salpingooophorectomy. The FS was then done on the specimen. If high-risk features were present on the FS, decision was taken to perform lymphnode dissection. Lymphadenectomy was also done on some low risk cases, based on the treating physician's discretion. The inclusion criteria were: endometrial cancer limited to uterus by clinical assessment & imaging studies, low grade histology by preoperative endometrial biopsy or dilation and curettage, and low grade (grade I & grade II) endometrial cancer by intra operative FS with none or <50% myometrial invasion. Exclusion criteria were: intra-operative FS findings of grade III, lymphovascular space invasion (LVSI), poor prognosis histologic type like carcinosarcoma, serous papillary or clear cell cancer, patients with intra-operative FS that showed more than 50% depth of myometrial invasion and cases where extra uterine disease was identified during the surgery. Also patients with synchronous primary ovarian tumors were excluded.

Intraoperative FS assessment: the specimen was sectioned serially at 2–3 mm intervals, grossly examined and areas of maximum macroscopic depth of invasion were obtained for intra-operative assessment. Two sections were taken for FS analysis. For assessment of cervical involvement, two sections were obtained from the lower uterine segment and endocervical junction for FS assessment. A median of 4 sections were examined for each patient. The sections were reviewed by the on-call pathologist and reported to the surgeon. The maximum time from receiving the specimen to having the report was 20 minutes. All final pathology reports were determined by a gynecologic pathologist. For the study, the FS slides were also reviewed by a gynecologic pathologist together with the lymph node specimens without knowing the results of final pathology results. The agreement between the PS and FS was assessed using the agreement statistic, (kappa). Comparison between the categorical variables was assessed using the chi square test. A ‘p’ value of ≤0.05 was considered statistically significant. SPSS was the statistical software used for the analysis (SPSS Inc, Chicago, Ill).

## Results

A total of 457 cases of endometrial carcinoma were treated by hysterectomy during this study period at our institution of which 177 cases were evaluated for tumor grade and depth of myometrial invasion by intra-operative FS. At the time of FS analysis, 18% (31/177) patients were found to have a high grade (FIGO grade III) tumor or a poor prognosis histology type and were hence excluded; leaving 146 patients for the final analysis. The median age of the study cohort was 60 (range 24–88) years.

**Figure 1:**
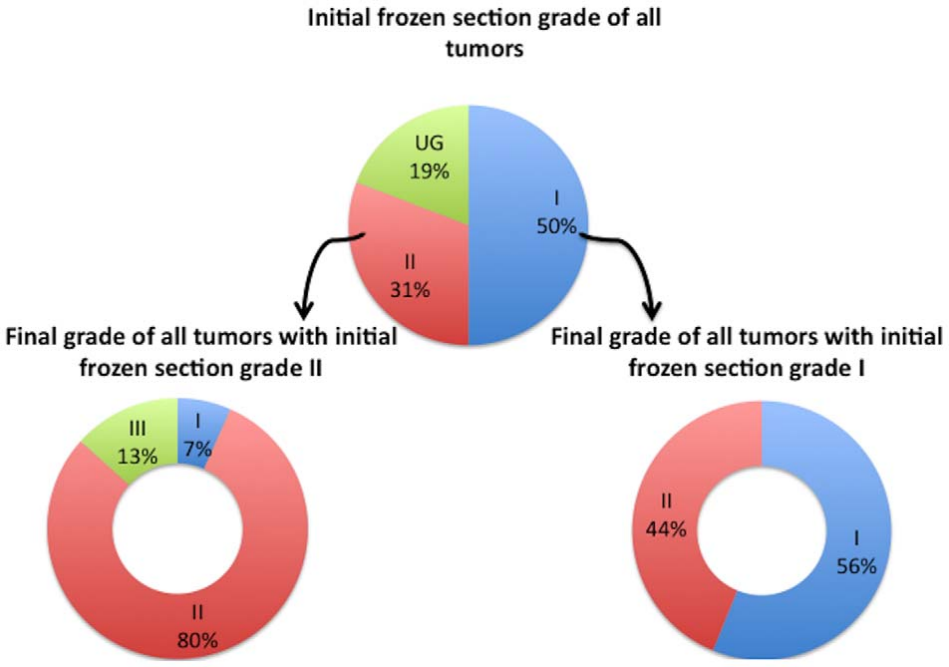
Relationship of tumor grade in frozen section to that of final pathology.

Of the entire study cohort (n = 146), 73 (50%) were grade I, 45 (31%) were grade II and 28 (19%) remained ungraded on initial FS. These ungraded cases are not included in table 1 and are represented by (UG) in figure 1. Of the 73 cases with grade I disease, the FS and PS were in agreement in 41 (56.2%) patients but the final pathology grade was advanced to grade II in 32 (43.8%, table 1, figure 1). For grade II by FS, 36 (80%) were correlated in the final pathology report, whereas 6 cases (13.3%) were upgraded to grade III & three (6.7%) cases were downgraded to grade I (table 1, figure 1). Concordance between frozen and permanent section for assessment of grade was 65.3% (kappa 0.58, 95%CI 0.51–0.68).

**Table 1:** Comparison of histologic grade in frozen and permanent sections.

Frozen section	Permanent section diagnosis		
Grade 173	Grade 141(56.2%)	Grade 232(43.8%)	Grade 30
Grade 245	Grade 13(6.7%)	Grade 236(80%)	Grade 36(13.3)

Myometrial invasion was assessed in 146 patients. By FS, 47 were found to have no MI (FS stage 1A as no extrauterine disease identified at FS or preoperatively). Of these 47, 32(68%) were in agreement on PS (table 2) whereas the remaining 15(32%) had varying degree of MI. On the other hand, 99 patients were reported to have <50% MI on FS (IFS stage 1B as no extrauterine disease identified at FS or preoperatively). Of these, 73(74%) were in agreement (64 with final stage 1B whereas 9 with final stage II –table 2). Of the remaining 26(26%), 12 had no MI whereas 14 were found to have ≥50% MI. Concordance between frozen and permanent section for assessment of myometrial invasion was (105/126) 72% (kappa 0.61, 95%CI 0.53–0.69).

**Table 2:** Comparison of stage in frozen and permanent sections.

Frozen section stage	Permanent section stage						
I A47	IA32(68%)	IB11(23.4%)	IC0	IIA1(2.1%)	IIB0	IIIA0	IIIC3(6.3%)
IB99	IA12(12%)	IB64(64.6%)	IC7(7%)	IIA7(7%)	IIB2(2%)	IIIA3(3%)	IIIC4(4%)

**Figure 2:**
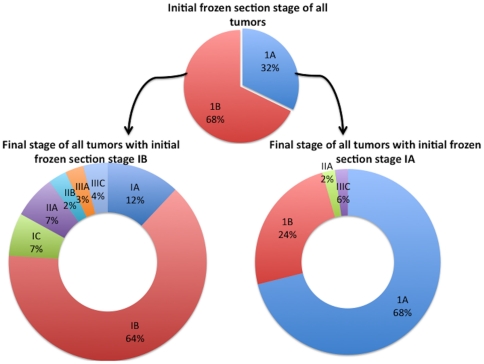
Relationship of FIGO stage in frozen section to that of final pathology.

Regarding the stage of the disease, 47 cases evaluated by FS were found to have stage IA disease of which 32 (68%) were in agreement, 11(23.4%) were upstaged to IB, 1 (2.1%) upstaged to IIA and 3(6.4%) upstaged to IIIC. On the other hand, 99 patients were thought to have stage IB disease by FS of which 64(64.6%) were in agreement, 12 (12.1%) down staged to IA, 7 (7%) upstaged to IC, 9 (9%) upstaged to II A & B (7% & 2% respectively), 3 (3%) upstaged to IIIA, and 4 (4%) upstaged to stage IIIC (table 2, figure 2). The FS displayed no cervical involvement for 122 patients in the study cohort of which 16 (13%) were determined to be false negative by the permanent histology by virtue of identification of tumor involving the cervix. Concordance between frozen and permanent section for assessment of cervical invasion was 86.9% (kappa 0.78, 95%CI 0.65–0.91) The FS stage in these 16 patients was FIGO IA in 3 and FIGO IB in 13. By definition of the inclusion criteria, all patients were negative for LVSI by FS. However, on final pathology report, 34 (31.7%) patients had LVSI, 73 did not have LVSI and in the rest 39, it was not assessed. It is noteworthy that 4 (14.2%) of patients with stage IA, and 30 (37.9%) of stage IB by FS, had positive LVSI. Concordance between frozen and permanent section for assessment of LVSI was 68.3% (kappa 0.60, 95%CI 0.52–0.69).

**Figure 3:**
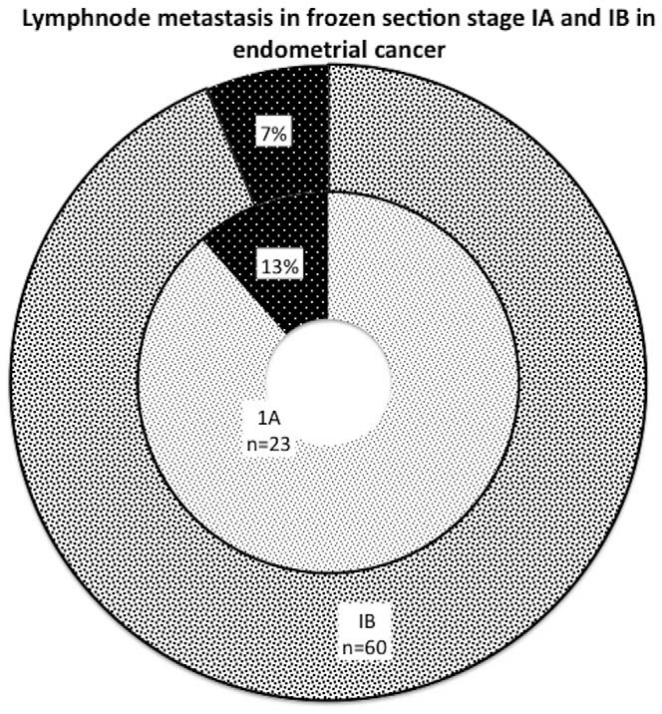
Lymphnode metastasis in frozen section FIGO stage IA & 1B.

A total of 83 of 146 (56.8%) patients underwent lymph node dissection. Of these patients, 97.6% underwent pelvic while 42.1% underwent paraaortic lymph node dissection. The median number of pelvic nodes obtained was 7 (range 1–35) and that of paraaortic lymph nodes was 4 (range 1–14). In all, 7 (8.4%) had lymph node metastasis. Pelvic lymph node metastasis was found in 5 patients (6%), whereas aortic lymph node metastasis was found in 1 (1.2%). One patient had involvement of both, pelvic and paraaortic nodes (1.2%). Of the 47 patients who had FS stage IA disease, lymphadenectomy was done in 23 (48.9%) while 24 patients did not have complete surgical staging. Of those who underwent lymphadenectomy, 3 (13%) cases had positive lymph nodes and were upstaged from IA to IIIC. All of these 3 cases had a change in myometrial invasion from none to a median of 22% (18%, 22% and 32% individually). In addition, they also had a change of FS grade 2 to PS grade 3. Of the 99 patients with FS stage IB disease, 60(60.6%) patients had lymphadenectomy and 39 cases did not. A total of 6.7%(n = 4/60) patients in this subgroup had positive nodes and were upstaged to FIGO Stage IIIC (figure 3). The median myometrial invasion for these 4 patients was 58%. The overall rate of agreement between the FS and that of the PS for variables in question (grade, myometrial invasion, lymphovascular space invasion and cervical invasion) is presented in table 3 along with the agreement statistic; kappa.

**Table 3:** (%) Agreement between frozen section and paraffin section with the corresponding agreement statistic (Kappa) for different variables in endometrial cancer.

Variable	(%) Agreement	Kappa	(95% Confidence Interval for Kappa)	p
Myometrial invasion	72	0.61	0.53–0.69	<0.0001
Cervical invasion	86.9	0.78	0.65–0.91	0.002
Lymphovascular space invasion	68.3	0.60	0.52–0.69	0.01
Grade	65.3	0.58	0.51–0.68	0.003

## Discussion

The management of low risk endometrial cancer has been a subject of great controversy in our times. Central to the controversy remain the independent yet intertwined issues of lymphadenectomy and postoperative radiation therapy. Unfortunately, rather than forging ahead with a unified theory of treatment of this disease, there has been an emerging dichotomy between the European and the North American way of management of low risk endometrial cancer. The European approach depicts treatment of low risk endometrial cancer by performing a total abdominal hysterectomy and bilateral salpingooophorectomy without dissection of lymphnodes routinely. This approach had been demonstrated by several prominent treatment centers participating in some of the largest prospective randomized control trial programs in endometrial cancer [9], [16]. The advocates of this approach cite a perceived lack of benefit from routine lymphadenectomy in low risk endometrial cancer [5] [6] along with achieving a potential benefit by sterilizing the nodal harbors of residual neoplastic clones in the lymphatic basins by aggressive use of adjuvant radiation [16]. In contrast is the North American approach advocating a routine lymph node assessment in endometrial cancer demonstrated by large cooperative group trial programs [10] and suggested by the American College of Obstetrics and Gynecology [17]. Supporters of this approach believe that surgical removal of lymphatic basins reduces tumor burden and minimizes the blanket use of unnecessary radiation, which will be given more frequently if surgical removal of lymphatic basins was not undertaken in this patient population. The gnawing limitations emanating from inadequate lymph node dissection and a lack of standard postoperative adjuvant treatment has introduced serious flaws in the prospective randomized lymphadenectomy trials [6] [5] and has further fuelled the controversy surrounding the role of lymphadenectomy in low risk endometrial cancer [18]. It has therefore become essential to examine an approach suggested by many as a reasonable compromise between the two polar opposite management strategies, namely; selective lymphnode dissection in low risk endometrial cancer based on high risk histology features discovered during intraoperative FS. This approach is based on the prerogative of maximizing pre-test probability of finding lymphnode metastasis if formal lymphadenectomy was undertaken and at least in theory, has the promise of avoiding the expense & morbidity of lymphadenectomy in patients who are at extremely low risk of lymphnode metastasis. On the other hand, it can also reduce the prescription of unnecessary blanket postoperative radiation. However, this intermediate approach of FS based selective lymphadenectomy in low risk endometrial cancer relies on two critical factors: the (%) agreement between FS and PS (because the historic risk factors for lymphnode assessment are based on final pathology rather than FS [4]) and the accuracy of these variables in predicting the actual lymphatic metastasis. In the present study, we evaluated the former of these two critical factors.

The first objective of this study was to correlate the grade and depth of myometrial invasion by FS with that of permanent pathology. Our results correlate with that of Frumovitz et al [14] who showed that FS analysis of tumor grade and depth of myometrial invasion are not always concordant with that of permanent sections. In the present study, for the intra-operative grade I, 44% were upgraded while in grade II, 13% were upgraded and 6.6% were downgraded. Hence in our series, there was 34.8% disagreement in assessing the grade of the tumor in comparison with PS. The clinical significance of upgrading in endometrial cancer was well depicted by Creasman et al. [4] in a seminal GOG study showing that a change of grade from I to II doubled the probability of middle third as well as outer third myometrial invasion; both of which signify a higher recurrence rate, poorer prognosis and generally call of additional adjuvant radiation [16] [19]. Along the same lines, we observed that in assessment of depth of myometrial invasion, disagreement was found in 28% of the cases in comparison with the PS with the overall agreement rate of 72%. More importantly, 7% of the cases were upstaged from FIGO stage IB to IC; a subgroup of endometrial cancer patients with extremely poor prognosis [20]. In this study, lymph node dissection was done in 56.8% of the lesions evaluated by FS and positive lymphnodes were found in 8.4% of them overall. Our data display that 13% and 6.6% patients in FS stage IA and IB respectively had lymphnode metastasis. The seemingly paradoxical finding of a higher rate of nodal metastasis in FS stage 1A in comparison to FS stage 1B can be explained by the fact that the three patients who had the nodal metastasis in FS stage 1A were all upgraded from grade 2 to grade 3 along with an amended extent of myometrial invasion from none (on FS) to a median of 22% (on PS). These data display that if FS was used in isolation for risk stratification; 7% to 13% patients would have received suboptimal treatment by forgoing lymphadenectomy as they would have had positive nodes on lymphadenectomy. Although the statistical measure of agreement (kappa-table 3) was generally in good/excellent range between the FS and PS; a 7%–13% prevalence of missed nodal metastasis seems clinically unacceptable for low risk endometrial cancer patients. Therefore, the interpretation of kappa in this specific scenario needs to be in context of the clinical implications rather than independent of the later. The risk of pelvic lymphnode involvement increases fivefold and that of paraaortic lymphnode involvement increases six fold as the depth of myometiral invasion changes from superficial to deep [4]. It is not unreasonable to expect that in routine clinical practice, disagreement of FS in prediction of grade, myometrial invasion and their cumulative impact on reducing the prevalence of surgical assessment of lymphnodes will be mutually multiplicative and has the potential of leading to the suboptimal treatment in a substantial number of patients. Similar to our study, others have reported a 5%–7% [12] [13] risk of suboptimal surgical treatment of endometrial cancer patients when FS analysis is considered as the basis of surgical management. The overall agreement on LVSI between FS and PS was 68.3%. The probability of finding LVSI on PS was higher for FS stage 1B as compared to 1A (p = 0.02, Chi square). This observation will be consistent with the known risk stratification value of LVSI where a presence of the same signifies a higher risk category of endometrial cancer.

Selection of a section of endomyometrium for assessment of the maximum depth of invasion is based on gross evaluation of the tumor. This might prove to be difficult as often the findings could be subtle especially when associated with a grade I tumor. Furthermore, determination of the exact extent of myometrial involvement in the setting of FS is challenging as the invasion line can be extremely heterogeneous with presence of skip metastasis as pointed out by others [12]. FS has poor sensitivity to detect microscopic foci of the disease in the cervix, which could be found only in permanent sections [21]. This was displayed in the present study where the FS was 13′% inaccurate on assessing the cervical involvement by the tumor in comparison to the permanent sections. The factors responsible for disagreement of FS grade include inadequate sampling to assess the amount of solid growth [22] and/or technical artifact associated with the surgery or FS process which might hamper the assessment of nuclear atypia. Evaluating additional sections would increase the agreement rate between the FS and PS however it will delay the time to diagnosis negating the advantage of this procedure [12]. Although we do not have data in support, but it seems likely that the errors in FS may be higher in absence of specialized gynecologic pathologists in many community hospitals. This would indirectly imply that where possible, referral to a gynecologic oncologist might be considered at the initial diagnosis of endometrial cancer because these physicians are specifically trained to perform accurate surgical staging of endometrial cancer.

Although the therapeutic value of lymphadenectomy is debatable in low risk endometrial cancer, the prognostic and treatment planning implications are clear [23]. Therefore, the consequences of errors that lead to incomplete surgical staging can be substantial. Patients who are incompletely staged at the time of the surgery and found to have high risk disease on final pathology, may need extended field radiotherapy or further staging procedures [24] [12]. The combination of surgery and radiotherapy is associated with significant morbidity in up to 12% of the patients [24]. Finally, the adverse financial impact of suboptimal surgical staging has been shown by many as well [24], [25], [26].

The Limitation of this study is its retrospective nature combined with limited number of patients. However the data was collected from a single cancer center and the FS slides were reviewed and compared with the permanent sections by a gynecologic pathologist who was blinded to the final outcome at the time of assessment of the FS. Therefore, the bias in assessment of FS histology was minimized. It is to be noted that the application of the new FIGO 2009 staging criteria of the endometrial cancer to our data may decrease the disagreement rate of the myometiral invasion between FS and PS in terms of the upstaging. This is because the new FIGO 2009 staging clubs together the older (1988) stage IA and 1B. However, a large study of prospectively maintained data at the Memorial Sloan Kettering Cancer Center, New York [27], has highlighted the limitation of the new staging schema as it eliminates the group of patients with the best prognosis (i.e. old stage 1A = no myometrial invasion). It is also correctly noted that the predictive value of the prognosis for the new stage 1 system represents no improvements in comparison to the older system. Therefore, our data is presented according to the FIGO 1988 staging scheme.

In summary, our data displays that FS is poor indicator of tumor grade and depth of myometrial invasion compared with that of final permanent section report. Significant number of patients with low-grade endometrial cancer based on intraoperative FS will have a higher grade, a higher stage and presence of lymphovascular space invasion on final pathology. Because a substantial number of patients in our study underwent a lymphnode dissection and were not identified to be at risk of lymphnode metastasis by FS, we learned that the wisdom of basing a decision of lymphadenectomy in endometrial cancer on the FS may lead to suboptimal treatment of these patients. Hence, as recommended by the American College of Obstetricians and Gynecologists (ACOG) [17] we support the notion of complete surgical staging along with lymphnode retrieval for patients with low grade early stage endometrial cancer unless medical contraindications exist.
